# Retinoblastoma: a curable, rare and deadly blinding disease

**Published:** 2018-06-03

**Authors:** Richard Bowman

**Affiliations:** 1 Honorary Clinical Consultant: International Centre for Eye Health, London School of Hygiene and Tropical Medicine, London, UK.

**Every year, thousands of babies and children in low- and middle-income countries lose their sight and their lives to a treatable childhood eye cancer called retinoblastoma; usually because it was not recognised and treated in time.**

**Figure:**
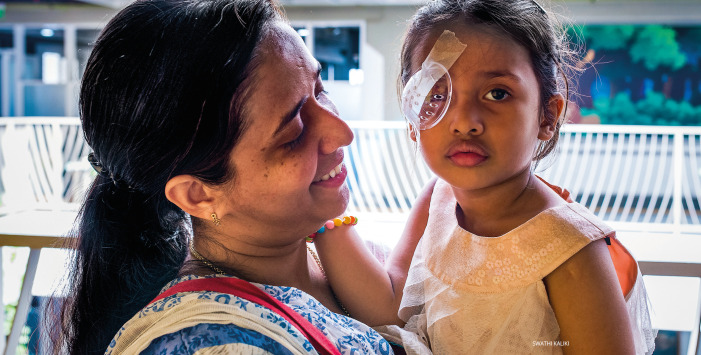
Early diagnosis and treatment can save the life of a child with retinoblastoma. INDIA

Although retinoblastoma is relatively uncommon, it can have devastating consequences for the children affected by it. If treated too late, it can lead to the loss of the eye, invasion of the brain and death.

Retinoblastoma does not affect everyone equally. In high-income countries, fewer than 5% of children die as a result of the condition, thanks to early diagnosis and specialist treatment. In Africa, however, it is typical to see 70% of children with retinoblastoma die, mainly because they presented too late. When mothers do present at a tertiary centre with a child who has advanced retinoblastoma, they often report that they have had several interactions with different health professionals over many months or even years, but did not get the referral or care they needed. Every health professional reading this issue of the *Community Eye Health Journal* has a chance to redress this balance. We need to find and treat children with retinoblastoma early, before it causes disfigurement or death.

Doing so successfully requires adopting a multidisciplinary, multi-level and internationally collaborative approach that looks at the health system as a whole (see page 4). Raising awareness of retinoblastoma in the community, improving the detection and diagnosis of the condition, setting up good referral systems and offering good counselling and high quality treatment (including good prosthetics) are all needed to increase the uptake of services and save lives.

## Ministry of health

Ministries of health have the power to made dramatic improvements to the early detection and treatment of retinoblastoma. They can:

- Create public health campaigns to raise awareness that ‘seeing something white’ inside a child's eye is a medical emergency
- Include basic ocular history taking and eye examination techniques in the curriculum of community nurses
- Offer subsidised access to specialist treatment for children with this life-threatening condition.

**Parents** must be made aware that they should seek help urgently if they see something white inside their child's eyes. Emphasise that parents should not let their child be turned away and must not take ‘no’ for an answer if they feel there is something wrong.

### A worthwhile investment

Investing resources in the early detection and referral of children with retinoblastoma has wider benefits in the fields of childhood blindness in low- and middle-income countries, as the same criteria (something white in the eye) will also help with the early detection of childhood cataract. Late presentation of childhood cataract is the leading cause of treatable blindness in children, and is entirely preventable if cataracts are detected and treated in time.

## In the community

Nurses and health workers seeing children in the community can check children's eyes during routine immunisation appointments, for example. **Something abnormal, white or shiny, or a squint, may be the first sign of retinoblastoma and requires urgent specialist referral.** Listen to the parents and/or carers. If they have seen something white or abnormal in their child's eye, believe what they say, take it seriously and seek specialist advice.

**Figure:**
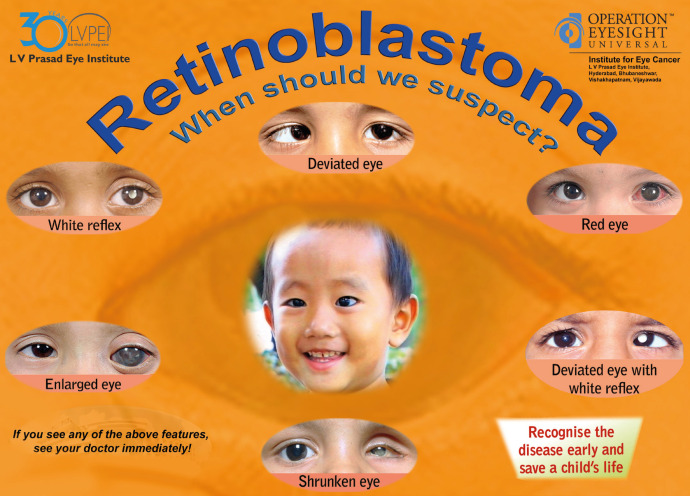
Public health awareness campaigns can support the early detection of retinoblastoma INDIA

In Tanzania, community nurses have been trained to examine the red reflex (p. 23) using an Arclight ophthalmoscope. The Arclight is an affordable, solar-powered and easy-to-use ophthalmoscope. It has shown preliminary promise; the community nurses found it easy to learn and began picking up cases of cataract and retinoblastoma by using it. Nurses can learn how to examine the red reflex at the same time as examining the child's other systems.

## Tertiary centres

At tertiary centres, histopathologists have a crucial role: once the eye is removed the child may be able to leave hospital completely cured or may need chemotherapy or radiotherapy; this decision must be based on accurate histopathological staging (page 18).

## International collaboration

To promote this multi-level, multi-disciplinary and internationally collaborative approach, the Commonwealth Eye Health Consortium has provided start-up funding for an Rb-Network known as Rb-NET, which has already generated specific country plans, a set of core outcome indicators, best practice protocols and a practical resource manual (**http://cehc.lshtm.ac.uk/dr-links/rbnet/**).

Basic clinical research questions still need to be answered. For instance, researchers in Uganda have shown an improvement in survival by giving chemotherapy before surgery on the basis that so many children have extra-ocular spread at time of presentation. On the other hand, a small study from Tanzania showed that 60% of children for whom there was good histology after enucleation had complete excision of the tumour with low risk and never needed chemotherapy. So which should come first in these settings - chemotherapy or surgery? By combining multi-centre and multi-country clinical research, as Rb-NET has started to do, we can begin to answer these questions and prevent needless tragedies.

This issue of the *Journal* demonstrates that there is real momentum and determination to improve outcomes for children with Rb in all countries across the world. It contains concise, practical information that should help all of us to make a difference.

**Table 1:** Roles and responsibilities in the detection, referral and treatment of retinoblastoma

Individual responsibilities			
Parent →	Health worker/nurse →	Ophthalmologist →	Specialist eye centre
Seek help urgently if you see something white inside the centre of the eye (the pupil) OR if you take a photograph and only one eye has a red dot in the centre Do not let anyone turn you away and do not take no for an answer until a doctor at a hospital has examined the child's eyes using a bright light	Believe the parents if they say they have seen something white inside the pupil and seek specialist advice. Treat it as a medical emergency Learn how to test the red reflex (p. 23). Test all children during routine visits and immunisations	Learn to recognise retinoblastoma and to identify eyes that need enucleation Counsel parents about the good cosmetic outcomes of enucleation with implantation. Show pictures of children with good outcomes Learn how to enucleate, taking more than 15 mm of optic nerve. Always examine the fundus of the fellow eye when you perform an enucleation: there could be a small tumour which is treatable by laser Refer all children with signs of retinoblastoma in two eyes to a national or specialist centre for urgent treatment	Same as for ophthalmologists, plus: Learn how to give focal or laser treatment to smaller tumours (usually in the second eye) Create multidisciplinary teams who work closely together to coordinate the treatment of each child Include in this team: ophthalmologists, oncologists, histopathologists, nurses, child life specialists or play therapists and/or counsellors Offer general and genetic counselling to parents/carers Refer parents to other sources of support for their child's learning and development
**The Ministry of Health's responsibilities towards the above**			
Run public awareness campaigns so that parents know that treatment is possible and know when to see a doctor	Ensure that the red reflex test (p. 23) is included in the curriculum for nurses and health workers	Ensure there is at least one ophthalmologist per 100,000 population	Support the development of national retinoblastoma centres and referral networks. Offer subsidised access to specialist treatment for all children with retinoblastoma. Provide screening services for siblings and accommodation or travel subsidies for the parents or carers of these young children.

**I am a child, not a case**

**Figure:**
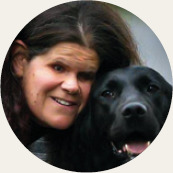
Abby White

Chief Executive: World Eye Cancer Hope UK **www.wechope.org**

Patients with retinoblastoma are first and foremost children who happen to have cancer in their eye(s). Defining a child as a ‘case’ dehumanises them and draws attention away from thoughts about their complete wellbeing and that of their family.

A child with retinoblastoma is not a medical specimen. They are a complete individual with thoughts, feelings, hopes, dreams, likes and dislikes. They have the ability to generate every kind of emotion in those who care for them. They are desperately loved, and most parents would give their own eye if it could spare their child's suffering.

In evaluating different treatments, we must weigh the value of each treatment in relation to the child's complete wellbeing. We must look beyond the physical body to embrace and care for the child's emotional health, during therapy and long after into adulthood.

Perhaps if we collectively take care to consider ‘children’, ‘families’ and ‘survivors’ rather than ‘cases’, we will together establish a level ground on which we can both treat the cancer, and heal the spirit in equal measure to set the child up for a healthy, happy future.

**Rb-NET website**

**http://cehc.lshtm.ac.uk/dr-links/rbnet/**

**Resource manual**

**http://cehc.lshtm.ac.uk/resource-manual-for-rb-management-v-sept-1-002/**

**Arclight**

**www.arclightscope.com**

